# A Double-Edged Impact of Social Smartphone Use on Smartphone Addiction: A Parallel Mediation Model

**DOI:** 10.3389/fpsyg.2022.808192

**Published:** 2022-02-07

**Authors:** Kuo Chang, Xue Li, Lei Zhang, Hui Zhang

**Affiliations:** School of Medical Humanities, Capital Medical University, Beijing, China

**Keywords:** smartphone addiction, social smartphone use, social support, agreeableness, online social support

## Abstract

Evidence supports predictive roles of non-social smartphone use for smartphone addiction, but the relationship of social smartphone use and smartphone addiction is unclear. This study explored whether social smartphone use has a double-edged impact on smartphone addiction. Using data from a sample of 909 Chinese undergraduates, we tested a parallel mediation model that considered online social support and realistic social support as mediators. As predicted, social smartphone use weakened smartphone addiction through realistic social support and contributed to smartphone addiction through online social support. Moreover, we tested the moderating role of agreeableness in the mediation path of online social support. Agreeableness only moderated the indirect effects. Specifically, the predictive effects of online social support on smartphone addiction was greater for lower rather than higher agreeableness. The results suggest that social needs play an important role in the formation of smartphone addiction. Several limitations and implications are also discussed herein.

## Introduction

Smartphones are used excessively worldwide to increase productivity and sociability and as ways to access various forms of entertainment (e.g., watching videos and playing games). Indeed, without smartphones, the world as we now know it would cease to exist. As indicated by a recent report, there are over 780 million smartphone users in China, and this number is increasing rapidly (CNNIC, 2018). With this increase in smartphone use, more research has been dedicated to the adverse outcomes of smartphone use. Previous research has considered smartphone addiction (also known as “smartphone overuse” and “problematic smartphone use”) to be a type of behavioral addiction, much like online game addiction (Kwon et al., 2013). Smartphone addiction is associated with psychopathology, including depression, anxiety, and stress (Kim et al., 2019; Sohn et al., 2019), body pain (Shan et al., 2013), emotional intelligence and self-regulation (Mascia et al., 2020), and poor sleep quality (Selçuk and Ayhan, 2019; Stanković et al., 2021). Given that smartphone use does not inevitably lead to detrimental outcomes and smartphone addiction, researchers have sought to clarify why some users become addicted to smartphones while others do not.

The development of smartphone addiction can be explained by Positive Reinforcement and Negative Reinforcement Theory (Elhai et al., 2016). To be specific, based on Use and Gratifications Theory (UGT; Blumler and Katz, 1974) and Compensatory Internet Use Theory (Kardefelt-Winther, 2014), researchers have proposed that smartphone addiction is a maladaptive strategy to pursue pleasure (UGT) and cope with negative emotions (Compensatory Internet Use Theory). Recent research has used a more comprehensive approach, namely, the Interaction of Person-Affect-Cognition-Execution model (Brand et al., 2014, 2016), to study smartphone addiction. The model takes predisposing variables (e.g., personality and biopsychological constitution), affective and cognitive responses, and executive and inhibitory control behaviors into account to explain Internet addiction. New findings have indicated that a dysfunctional coping strategy (e.g., expressive suppression), the type of smartphone use (e.g., non-social smartphone use), and the specific type of anxiety (e.g., a fear of missing out) can predict smartphone addiction (Elhai et al., 2018, 2020; Rozgonjuk and Elhai, 2019). Furthermore, social support as a social factor has been reported to be negatively correlated with smartphone addiction (Herrero et al., 2019a) and can negatively predict smartphone addiction (Herrero et al., 2019b).

Although previous studies have deepened our understanding of smartphone addiction, results concerning the relationship between social smartphone use and smartphone addiction were inconsistent. Some studies indicated that social smartphone use might predict smartphone addiction. A significant positive correlation between social smartphone use and smartphone addiction was found in studies conducted by Rozgonjuk and Elhai (2019) and Elhai et al. (2020). Rozgonjuk et al. (2019) found that social smartphone use could significantly predict smartphone addiction. As a typical social use of smartphones, Facebook use has been found to enhance online social support (Liu and Yu, 2013). Online social support may have the same function as realistic social support (the care and support people feel or receive from real life), and can play a beneficial role (Cheung et al., 2017) or increase the risk of Internet addiction and smartphone addiction (Ding et al., 2013; Zhao et al., 2021). Others indicated that social smartphone use might not predict smartphone addiction. van Deursen et al.’s (2015) study, social smartphone use could not predict smartphone addiction. It is not yet clear whether people with more social use of smartphone tend to be addicted to smartphones, or whether online social support plays the same role as realistic social support in the relationship between smartphone use and smartphone addiction. The purpose of the present study was to clarify the relationship between social use of smartphones and smartphone addiction, and the internal mediating mechanism.

### Social Use of Smartphones and Smartphone Addiction

Technology feature use has been divided into process and social use (Song et al., 2004). Social use of smartphones refers to the use of smartphones for social purposes, such as using social media or maintaining social relationships; process use of smartphones refers to the use of smartphones for entertainment, relaxation, and other non-social purposes (Elhai et al., 2017). Social use of smartphone creates a unique space in which people can share their life and make new friends without face-to-face communication, which can reduce social anxiety and enhance social comfort.

Indulging in such use can cause people to become addicted to smartphones. For example, abuse of Facebook may lead to Facebook addiction (a kind of social networking addiction) and the broadcasting behavior on Facebook, Facebook using intensity could predict Facebook addiction (Ryan et al., 2014; Xie and Karan, 2019). Studies on Internet addiction have shown that social use of the Internet is positively correlated with Internet addiction (Chou and Hsiao, 2000; Yang and Tung, 2007). As a behavioral addiction, smartphone addiction is similar to Facebook and Internet addiction, which can explain why similar results on smartphone addiction have been reported. Researchers have found that social media used together with smartphone gaming can predict smartphone addiction (Haug et al., 2015; Cha and Seo, 2018; Laurence et al., 2020).

Social use of smartphones may be beneficial and might buffer smartphone addiction, however. For instance, social use of smartphones promotes social engagement (Pendry and Salvatore, 2015), improves social skills, and increases social capital (Tsitsika et al., 2014). The use of social media may have social benefits, leading to enhanced life satisfaction (Zhan et al., 2016). The social use of smartphones could provide a buffer to people who are not comfortable with face-to-face socializing, and might allow them to engage in interpersonal relationships that may reduce smartphone addiction (Hong et al., 2019). Specifically, Facebook can be used to seek friendship and improve social support (Egbert et al., 2021), alleviate depressed mood (Frison and Eggermont, 2016), and help people to quit smoking (Borrelli et al., 2021). Social support is related to coping style (van Rijen et al., 2004; Wang et al., 2018), whereby people with high level of social support will adopt a positive coping style (Chen et al., 2019) rather than a negative coping style such as smartphone overuse. These double-edged impacts of social smartphone use on smartphone addiction are likely to be mediated by different factors.

### The Mediation Effects of Online Social Support and Realistic Social Support

It is not yet clear how social use of smartphones influences smartphone addiction. From the perspective of Maslow’s hierarchy of needs (Lester et al., 1983), the present study considered social support as a potential mediator and tested its mediation effect in the relationship between social use of smartphones and smartphone addiction. Social support refers to the care and support people feel or receive from others, and can reduce the adverse effects of negative factors on general wellbeing (Cohen and Wills, 1985). People with good social support have more resilience (Wang et al., 2019) and are better able to handle stressful life events (Panesar et al., 2021) instead of avoiding them. Maslow’s hierarchy of needs states that social needs dictate human behavior (Lester et al., 1983). Most of us need to be connected with others and to be respected, and these needs can be fulfilled by social support. Social use of smartphones can help people obtain higher levels of social support through the use of social media and social network sites (Lee and Cho, 2019; Wu and Chiou, 2020). Furthermore, social support has universal benefits and can help people overcome Internet addiction (Mo et al., 2018; Jung et al., 2019).

Online social support refers to the communication that occurs in virtual space, where people can feel understood and respected, and can receive emotions, information, and material support, thus enhancing identity and a sense of belonging (Nick et al., 2018; Zhao et al., 2021). To distinguish between the two kinds of social support, the present study defined realistic social support as the care and support people feel or actually receive from others. Compared with realistic social support, online social support is mainly received *via* the Internet, and may have different function. Although social media use can enhance online social support (Cole et al., 2017), it appears to have only a small direct effect on wellbeing (Liu and Yu, 2013). For example, one study revealed that only real-world social support was associated with reduced depression and anxiety, while in-game social support was unrelated to both (Tham et al., 2020). According to UGT (Blumler and Katz, 1974), people will be addicted to the behavior (e.g., surf the Net) which brings them gratifications. So, people who use smartphones to feel that they have strong social support and do not care whether it is real may become addicted to smartphones.

### The Moderating Effect of Agreeableness

From the perspective of the Interaction of Person-Affect-Cognition-Execution model (Brand et al., 2016), personality is a predisposing variable that can influence addiction behavior. It is not yet known whether the relationship between social use of smartphones, online social support, and smartphone addiction is moderated by personality, especially the positive effects of personality traits such as agreeableness. Agreeableness is a major dimension of personality (Graziano and Tobin, 2002) that refers to being considerate, friendly, helpful, and willing to give up one’s own interests for others. One study has shown that agreeableness influences aggression in adolescents (Gleason et al., 2004) and is associated with the ability to maintain positive interpersonal relationships in adolescents and adults (Jensen-Campbell et al., 2003).

The effect of agreeableness can be explained by UGT (Blumler and Katz, 1974). Namely, people often use the Internet to get what they lack in the real world. People with low agreeableness, for example, may have poor interpersonal relationships, sand feel isolated. To meet their needs of social contact, people with low agreeableness may use smartphones to receive online social support, because online social support is more convenient and easier to get than realistic social support. Furthermore, agreeableness is correlated with addiction problems; numerous studies have reported that agreeableness is negatively correlated with substance or behavioral addictions (Andreassen et al., 2013; Zilberman et al., 2018). Thus, low agreeableness may strengthen the impact of social smartphone use and online social support on smartphone addiction. People with high agreeableness receive good social support, adopt a positive coping style, and achieve a high level of life satisfaction (Agbaria and Mokh, 2021; Fors Connolly and Johansson Sevä, 2021), and so might not attach as much importance to social support as do people with low agreeableness. Therefore, we can predict that people with high agreeableness do not overuse smartphones for social networking, nor do they indulge in smartphones. Studies have supported the hypothesis that agreeableness is negatively correlated with smartphone addiction (Volungis et al., 2020). Thus, high agreeableness may weaken the impact of social smartphone use and online social support on smartphone addiction.

### The Present Study

**FIGURE 1:**
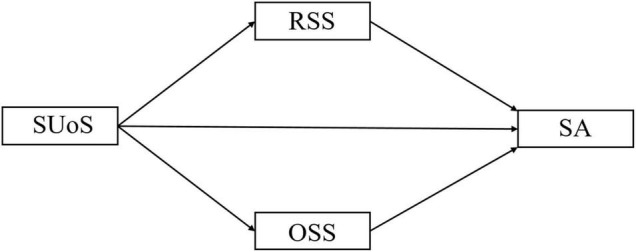
Hypothesis 1. SUoS, social use of smartphones; RSS, realistic social support; OSS, online social support; SA, smartphone addiction.

**FIGURE 2:**
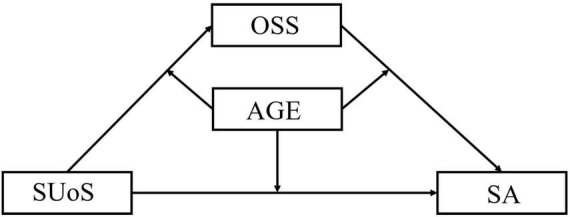
Hypothesis 2. SUoS, social use of smartphones; AGR, agreeableness; OSS, online social support; SA, smartphone addiction.

The main purpose of this study was to explore the relationship between social smartphone use and smartphone addiction, and test the parallel mediation effects of online social support and realistic social support in this relationship in Chinese undergraduates. Based on the aforementioned studies, we hypothesized that the social use of smartphones weakens smartphone addiction through realistic social support and contributes to smartphone addiction through online social support, and that the indirect effect of online social support on this relationship is significantly larger than the indirect effect of realistic social support (Hypothesis 1). We also hypothesized that agreeableness moderates the relationship between social use of smartphones and smartphone addiction (Hypothesis 2). These hypotheses are presented in Figures 1, 2.

## Materials and Methods

### Participants

Stratified cluster-sampling method was applied. First, we randomly selected four universities in Beijing, China, then we randomly selected four classes in each grade and distributed online questionnaires to all students in each class in the online class. We included undergraduate students who had at least one smartphone in this study. We asked the teachers to inform the students that our survey does not include students who are taking psychotropic drugs or receiving psychological counseling, or students with physical disabilities (e.g., blindness) who cannot complete the questionnaire and use smartphone alone. Finally, we got 909 participants (41.3% of the participants were male). These included 241 first-year students (26.5%), 257 sophomores (28.3%), 229 juniors (25.2%), and 182 seniors (20%). The mean age of the participants was 20.21 years (SD = 1.27) and their ages ranged from 17 to 25 years.

### Measures

#### Socio-Demographics

We collected information about participants’ age, gender, and grade, and participants completed several scales.

#### The Smartphone Addiction Scale for College Students

College students’ smartphone addiction was measured using the Smartphone Addiction Scale for College Students (SAS-C). The 22-item SAS-C was developed using data from Chinese undergraduate students. It includes items such as, “I feel the need to spend more time on my phone to be satisfied,” which are scored using a five-point Likert scale, with responses ranging from 1 (strongly disagree) to 5 (strongly agree). Higher total scores indicate a stronger addiction to smartphones (Su et al., 2014). The SAS-C has the six following subscales: “withdrawal behavior,” “salience behavior,” “social comfort,” “negative effects,” “use of application,” and “renewal of application.” The SAS-C can also be used as a unidimensional measure, and has very good internal reliability [Cronbach’s α = 0.88 in Su et al. (2014)]. In the present study, the measure had a good reliability (Cronbach’s α = 0.92).

#### Social Support Scale for University Students

College students’ realistic social support was measured using the Social Support Scale for University Students (SSS-U). The SSS-U was developed using data from Chinese college students, and has 17 items (e.g., “I often get care and support from my classmates and friends”) that are scored using a five-point Likert scale ranging from 1 (strongly disagree) to 5 (strongly agree). Higher total scores indicate greater realistic social support (Ye and Dai, 2008). The SSS-U consists of three subscales, including subjective support, objective support, support utilization, and can be used as a unidimensional measure. The scale has a good reliability [Cronbach’s α = 0.91 in Ye and Dai (2008), Cronbach’s α = 0.92 in the present study].

#### Online Social Support Scale

College students’ online social support was measured using the Online Social Support Scale (OSSS). The OSSS was developed using data from Chinese high school and college students. The OSSS has 23 items (e.g., “When I feel lonely, I can tell others through the Internet”) that are scored using a five-point Likert scale ranging from 1 (strongly disagree) to 5 (strongly agree), whereby higher total scores indicate greater online social support (Liang, 2014). The OSSS has the four following subscales: “friend support,” “emotional support,” “information support,” and “tool support.” The scale has a good reliability [Cronbach’s α = 0.91 in Liang (2014), Cronbach’s α = 0.91 in the present study].

#### Process vs. Social Smartphone Usage Scale

College students’ social use of smartphone was assessed using the social use subscale of the Process vs. Social Smartphone Usage Scale (PSSU). The PSSU has two subscales that measure process smartphone use (e.g., for entertainment) and social smartphone use (e.g., for social networking), respectively. This study used the PSSU social use subscale, which includes five items (e.g., “I use my smartphone to call other people”) that are scored on a five-point Likert scale ranging from 1 (strongly disagree) to 5 (strongly agree), whereby higher scores indicate more social use. The original Cronbach’s α = 0.73 (van Deursen et al., 2015), and in this study, the scale had a good reliability (Cronbach’s α = 0.71).

#### Neuroticism Extraversion Openness Five-Factor Inventory

The agreeableness subscale of the Neuroticism Extraversion Openness Five-Factor Inventory was used to measure college students’ agreeableness. This subscale has 12 items (e.g., “I would rather cooperate with others than compete with others”) that are scored on a five-point Likert scale ranging from 1 (strongly disagree) to 5 (strongly agree), whereby higher total scores indicate a higher level of agreeableness (Yao and Liang, 2010). In this study, the scale had good reliability (Cronbach’s α = 0.71).

### Procedure

The study protocol was approved by the Academic Committee of Capital Medical University. We collected data using an online questionnaire between March and June, 2021. We conducted the survey on a professional platform in China what is named “Wenjuanxing”.^1^ Informed consent was obtained before the formal questionnaire, and participants could choose whether to continue to participate in the survey after reading the study information. Participants received 5 RMB after they had completed all questionnaires.

### Statistical Analyses

All data were analyzed using SPSS 23.0 software for Windows. First, we tested the common bias in our study using Harman’s single-factor test, because the data were collected by self-reports. Second, we calculated the descriptive statistics and zero-order correlation matrix. The *t*-test was applied to test between-sex differences in the social use of smartphones, realistic social support, smartphone addiction, online social support, agreeableness, and one-way ANOVA (analysis of variance) was applied to test such differences by grade. We standardized the data before verifying the proposed model. Third, we built a parallel mediation model in which the SAS-C score was treated as the outcome, both the SSS-U and OSS scores were treated as mediators, and the PSSU social use subscale score represented the independent variable. The PROCESS Model 4 of SPSS was used to test the parallel mediation model. Finally, we used the PROCESS Model 59 to test the moderating effect of agreeableness in both the direct and indirect relationships between social use of smartphones and smartphone addiction, with online social support as a mediator. The bootstrap confidence intervals (CIs) were calculated based on 5,000 random samples to estimate the significance of the effects. The effect is considered significant when CIs do not contain zero (Hayes, 2013).

## Results

### Primary Analyses

Harman’s single-factor test based on exploratory factor analysis retained 16 factors with eigenvalues larger than 1, of which the first factor accounted for 16.58% of total variance, which was far less than 40% (Richardson et al., 2009). The results showed that common method bias was negligible in this study. The means, SDs, and correlations between the study variables are presented in Table 1. Social use of smartphones was positively correlated with realistic social support, online social support, and smartphone addiction. Realistic social support was positively correlated with online social support and agreeableness. Online social support was positively correlated with smartphone addiction and agreeableness was negatively correlated with smartphone addiction.

**TABLE 1:** Means, SDs, and Pearson’s correlation coefficients between the study variables (*n* = 909).

	1	2	3	4	5
1. Social use of smartphones	–				
2. Realistic social support	0.41**	–			
3. Online social support	0.37**	0.50**	–		
4. Smartphone addiction	0.11**	−0.01	0.28**	–	
5. Agreeableness	0.01	0.23**	−0.05	−0.50**	–
*M*	18.78	66.17	83.25	69.84	39.49
SD	3.50	10.90	14.80	16.85	6.62

*p < 0.05, **p < 0.01.

There were no significant between-sex differences in the social use of smartphones, realistic social support, smartphone addiction, or online social support, but women had significantly higher agreeableness scores than men (*t* = 4.89, *p* < 0.001, *d* = 0.26). Significant differences by grade were found for online social support, smartphone addiction, and agreeableness. Juniors had higher online social support scores (*F* = 6.54, *p* < 0.001) and smartphone addiction scores (*F* = 3.93, *p* < 0.01), and significantly lower agreeableness scores (*F* = 2.95, *p* < 0.05) compared with students in other grades.

### Parallel Mediation Model Test

**Hypothesis 1** proposed that social use of smartphones weakens smartphone addiction through realistic social support and contributes to smartphone addiction through online social support. In addition, we hypothesized that the indirect effect through online social support is significantly larger than the indirect effect through realistic social support because studies found that social smartphone use was positively correlated with smartphone addiction (van Deursen et al., 2015; Rozgonjuk et al., 2019; Rozgonjuk and Elhai, 2019; Elhai et al., 2020).

**FIGURE 3:**
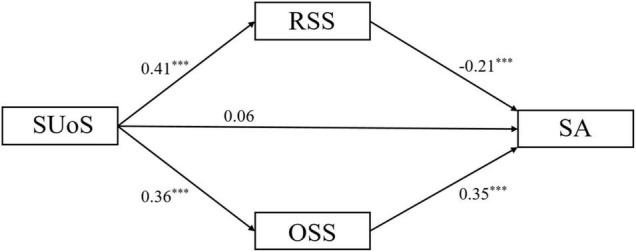
The standardized path coefficients of the parallel mediation model. SUoS, social use of smartphones; RSS, realistic social support; OSS, online social support; SA, smartphone addiction. ***p < 0.001.

A parallel mediation model was built taking age, sex, and grade as covariates. As shown in Figure 3, social use of smartphones was positively associated with realistic social support (β = 0.41, *p* < 0.001), and realistic social support was negatively associated with smartphone addiction (β = −0.21, *p* < 0.001). Social use of smartphones weakened smartphone addiction through realistic social support [indirect 1 = −0.08, CIs = (−0.12, −0.05)]. Social use of smartphones was positively associated with online social support (β = 0.36, *p* < 0.001), and online social support was positively associated with smartphone addiction (β = 0.35, *p* < 0.001). Social use of smartphones contributed to smartphone addiction through online social support [indirect 2 = 0.13, CIs = (0.09, 0.17)]. The difference of indirect 1 and indirect 2 was significant [indirect 2 – indirect 1 = 0.21, CIs = (0.16, 0.28)]. The *R*^2^ of parallel mediation model is 0.11. Thus, Hypothesis 1 was supported.

### Moderated Mediation Model Test

**FIGURE 4:**
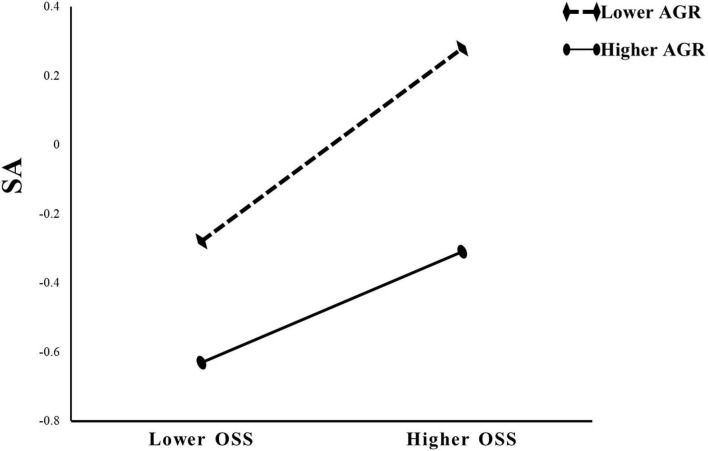
Moderation effect of AGR in the relationship between OSS and SA. AGR, agreeableness; OSS, online social support; SA, smartphone addiction.

**Hypothesis 2** was that agreeableness moderated the paths between social use of smartphones and smartphone addiction, with online social support as a mediator. The assumption was tested by the PROCESS Model 59 taking age, sex, and grade as covariates. As shown in Table 2, we firstly used social use of smartphones, agreeableness, and social use of smartphones × agreeableness to predict smartphone addiction, in model 1. The effect of social use of smartphones on smartphone addiction was not moderated by agreeableness (β = −0.01, *p* > 0.05). We secondly used social use of smartphones, agreeableness, and social use of smartphones × agreeableness to predict online social support, in model 2. The effect of social use of smartphones on online social support was not moderated by agreeableness (β = 0, *p* > 0.05). Finally, we used social use of smartphones, agreeableness, online social support, social use of smartphones × agreeableness, online social support × agreeableness to predict smartphone addiction, in model 3. Results showed that the effect of online social support on smartphone addiction was moderated by agreeableness (β = −0.12, *p* < 0.001), but social use of smartphones was not moderated by agreeableness (β = 0.004, *p* > 0.05). Furthermore, as shown in Figure 4, compared with online social support (β = 0.28, *p* < 0.001), agreeableness (β = −0.47, *p* < 0.001) had a greater main effect. The relationship between online social support and smartphone addiction was stronger when students had a low (1 SD below the mean) level of agreeableness [β = 0.40, SE = 0.05, *p* < 0.001, CIs = (0.31, 0.49)] than when students had a high (1 SD above the mean) level of agreeableness [β = 0.16, SE = 0.04, *p* < 0.001, CIs = (0.08, 0.23)]. Thus, Hypothesis 2 was supported.

**TABLE 2:** Testing the moderating effect of agreeableness on smartphone addiction.

Predictor	Model 1 (SA)		Model 2 (OSS)		Model 3 (SA)	
	β	*t*	β	*t*	β	*t*
SUoS	0.11	3.95***	0.37	11.72***	0.01	0.35
AGR	–0.50	−17.07***	–0.04	–1.38	–0.47	−16.60***
SUoS × AGR	–0.01	–0.33	0	–0.12	0.04	1.26
OSS					0.28	9.13***
OSS × AGR					–0.12	−4.16***
*R* ^2^	0.27		0.14		0.34	
*F*	56.31***		25.07***		57.09***	

SUoS, social use of smartphone; OSS, online social support; SA, smartphone addiction; AGR, agreeableness. ***p < 0.001.

## Discussion

The main purpose of present study was to explore the relationship between social smartphone use and smartphone addiction by testing the parallel mediation of online social support and realistic social support in Chinese undergraduates. Social smartphone use was positively associated with smartphone addiction, which was consistent with previous studies (van Deursen et al., 2015; Rozgonjuk and Elhai, 2019; Rozgonjuk et al., 2019; Elhai et al., 2020). We also found that the relationship between social smartphone use and smartphone addiction was parallel mediated by online social support and realistic social support. The path mediated by online social support contributed to smartphone addiction, while the path mediated by realistic social support weakened it, and the effect of former path was significantly larger than the latter. Therefore, the hypothesized parallel mediating model in this study was supported. Moreover, the present study tested the moderation effect of agreeableness in the path mediated by online social support. We found a moderating effect of agreeableness in the relationship between online social support and smartphone addiction. The present study implicates that besides pleasure needs, social needs play an important role in the formation of smartphone addiction and enhancing agreeableness might prevent smartphone addiction.

First, this study divided the concept of social support into online social support and realistic social support to explore the double-edged impact of social smartphone use on smartphone addiction. Then, the study found that online social support and realistic social support had different effects on the relationship between social smartphone use and smartphone addiction. On the one hand, social use of smartphones weakened smartphone addiction through enhancing realistic social support. This result is consistent with those of previous studies. For instance, people who use smartphones have been reported to receive more realistic social support (Oh et al., 2014). Social use of smartphones might promote people to establish realistic interpersonal relationships and increases the possibility of realistic social interaction, such as dinner appointments, dating, and sports. These relationships provide realistic social support. Furthermore, realistic social support has been reported to weaken smartphone and Internet addiction (Herrero et al., 2019b; Jung et al., 2019); this could be because people with more realistic social support tend to have a greater resilience (Wang et al., 2019) and to adopt positive coping strategies (Panesar et al., 2021). On the other hand, social use of smartphones contributed to smartphone addiction through pursuing online social support. Social media use might enhance online social support (Cole et al., 2017), but this form of social support does not have the same function as realistic social support (Liu and Yu, 2013); on the contrary, online social support can contribute to behavioral addiction, such as Facebook addiction (Xie and Karan, 2019). Social anxiety is associated with smartphone addiction (Wolniewicz et al., 2018), because social anxiety might promote people use their smartphones for online social networking to get high online social support and then online social support leads to smartphone addiction. It is worth noting that the indirect effect through online social support was significantly larger than the indirect effect through realistic social support. In other words, considering the indirect effect of both online social support and realistic social support, social use of smartphones should positively predict smartphone addiction. This is in line with previous findings that the social use of smartphones was positively correlated with smartphone addiction (Elhai et al., 2018, 2020; Rozgonjuk and Elhai, 2019).

Second, we tested the moderation effect of agreeableness in the relationship between social-use of smartphones and smartphone addiction. Agreeableness moderated the effect of online social support and not of social smartphone use. This indicated that the social-use of smartphones enhances online social support, while online social support can lead to smartphone addiction. We found that individuals with higher levels of agreeableness were less likely to become addicted to smartphones than those with lower levels of agreeableness. The buffer effect of higher agreeableness levels can be explained by Maslow’s hierarchy of needs theory (Lester et al., 1983) and UGT (Blumler and Katz, 1974). In accordance with Maslow’s hierarchy of needs theory, people generally have the need to feel connected with others. However, people with low levels of agreeableness may have social anxiety (Kaplan et al., 2015), which may mean their social needs cannot be met (Lee-Won et al., 2015). Therefore they use smartphones to satisfy their social needs while avoiding face-to-face communication. Based on UGT, pursuing positive experience or avoiding negative experience is an important reason for addiction. The gratification of online socializing might have different emotional titers for people within different levels of agreeableness. One study has reported that agreeableness is negatively correlated with social anxiety (Łakuta, 2019). Thus, people with high levels of agreeableness are more likely to have good personal relationships, both on the Internet and in real life. So, they do not regard online social support as the only way to meet social needs and less likely to indulge in smartphone use for social purposes. While people with low levels of agreeableness might have poor personal relationships and think highly of gratification of online socializing, leading to smartphone addiction. We found no moderation effect of agreeableness on social smartphone use in this study. Probably because social smartphone use is a common function of smartphones that increases social distance and avoids social anxiety, and can thus increase online social support, regardless of agreeableness. It is worth noting that the main effect of agreeableness on smartphone addiction was greater than online social support. This is consistent with previous study that showed that personality had a strong predictive effect on smartphone addiction (Brand et al., 2016). Given the benefits of online social support, such as emotions, information, and material support (Nick et al., 2018), a healthy approach could be to weaken the association between online social support and smartphone addiction, such as, making online social networking the first step of offline communication, turning net friends into friends in real life.

The current study has both theoretical and applied research significance in the study of smartphone addiction. Theoretically, this study contributes to the field by showing how social smartphone use linked to smartphone addiction. Previous studies emphasized the prediction of non-social smartphone use to smartphone addiction but ignored role of social smartphone use. The present study explored the predictive role of social smartphone use to smartphone addiction using online social support and realistic social support as mediator. We found that social use of smartphones weakened smartphone addiction through realistic social support and contributed to smartphone addiction through online social support. The present study provide evidence that social smartphone use has double-edged impact on smartphone addiction and social needs play an important role in the formation of smartphone addiction. Practically, our findings could contribute to future studies on smartphone addiction prevention and interventions. Given social smartphone use is a common function of smartphone, we do not encourage prevention in smartphone addiction by reducing smartphone use. The present study shows that realistic social support has beneficial effects and smartphone should be encouraged to enhance realistic social support, such as using smartphones to make more friends and turn them into real friends, not just net friends. Moreover, reducing online social support and convert online social support into realistic one is a good strategy to prevent smartphone addiction, especially for those who on low level of agreeableness.

This study has several limitations. First, we only measured social smartphone use without measuring non-social smartphone use, which meant that we could not examine whether non-social smartphone use contribute to smartphone addiction through online social support. We did not collect information about economic status and marital status of participants. Further studies should thus consider both social smartphone use and non-social smartphone use to understand the mechanisms underlying smartphone addiction and collect adequate information about subject characteristics. Second, we divided the concept of social support into online social support and realistic social support; however, these two concepts overlap to a certain degree. So, the stable negative correlation between realistic social support and smartphone addiction in previous studies (Rozgonjuk and Elhai, 2019; Elhai et al., 2020) was not found in our study. Future research should consider the differences between online social support and realistic social support in more detail. Third, the scales used in our study was developed based on Chinese sample and participants in this study are Chinese. Whether the research conclusions can be applied to people in other cultures remains to be further studied. Moreover, data were collected using a self-report method and the study had a cross-sectional design. Thus, common method bias may exist in this study, and the causal relationships between variables could not be determined. Future research should apply a longitudinal approach to further test the model proposed in this study.

## Conclusion

In conclusion, this study found a double-edged impact of social smartphone use on smartphone addiction. Social use of smartphones weakened smartphone addiction through realistic social support and contributed to smartphone addiction through online social support. High level of agreeableness will weaken the association between online social support and smartphone addiction.

## Data Availability Statement

The original contributions presented in the study are included in the article/supplementary material, further inquiries can be directed to the corresponding author.

## Ethics Statement

The studies involving human participants were reviewed and approved by the Academic Committee of Capital Medical University. The patients/participants provided their written informed consent to participate in this study.

## Author Contributions

KC conceived and designed this study, collected and analyzed the data, written, and revised the manuscript. XL, LZ, and HZ revised the manuscript. KC and HZ were responsible for the study. All authors contributed to the article and approved the submitted version.

## Conflict of Interest

The authors declare that the research was conducted in the absence of any commercial or financial relationships that could be construed as a potential conflict of interest.

## Publisher’s Note

All claims expressed in this article are solely those of the authors and do not necessarily represent those of their affiliated organizations, or those of the publisher, the editors and the reviewers. Any product that may be evaluated in this article, or claim that may be made by its manufacturer, is not guaranteed or endorsed by the publisher.

## Footnotes

1
https://www.wjx.cn/
